# Oncological healthcare providers’ perspectives on appropriate melanoma survivorship care: a qualitative focus group study

**DOI:** 10.1186/s12885-023-10759-9

**Published:** 2023-03-28

**Authors:** Nadia C. W. Kamminga, Marlies Wakkee, Rianne J. De Bruin, Astrid. A. M. van der Veldt, Arjen Joosse, Suzan W. I. Reeder, Peter W. Plaisier, Tamar Nijsten, Marjolein Lugtenberg

**Affiliations:** 1 Department of Dermatology, Erasmus MC Cancer Institute, University Medical Center Rotterdam, Rotterdam, The Netherlands; 2 Department of Medical Oncology, Erasmus MC Cancer Institute, University Medical Center Rotterdam, Rotterdam, The Netherlands; 3 Department of Radiology & Nuclear Medicine, Erasmus MC Cancer Institute, University Medical Center Rotterdam, Rotterdam, The Netherlands; 4 Department of Dermatology, Albert Schweitzer Hospital, Dordrecht, The Netherlands; 5 Department of Surgical Oncology, Albert Schweitzer Hospital, Dordrecht, The Netherlands; 6 Department Tranzo, Tilburg School of Social and Behavioral Sciences, Tilburg University, Tilburg, The Netherlands

**Keywords:** Melanoma, Survivorship care, Survivorship care plan, Qualitative research

## Abstract

**Background:**

The increasing group of melanoma survivors reports multiple unmet needs regarding survivorship care (SSC). To optimise melanoma SSC, it is crucial to take into account the perspectives of oncological healthcare providers (HCPs) in addition to those of patients. The aim of this study is to gain an in-depth understanding of HCPs’ perspectives on appropriate melanoma SSC.

**Methods:**

Four online focus groups were conducted with mixed samples of oncological HCPs (dermatologists, surgeons, oncologists, oncological nurse practitioners, support counsellors and general practitioners) (total *n* = 23). A topic guide was used to structure the discussions, focusing on perspectives on both SSC and survivorship care plans (SCPs). All focus groups were recorded, transcribed verbatim, and subjected to an elaborate thematic content analysis.

**Results:**

Regarding SSC, HCPs considered the current offer minimal and stressed the need for broader personalised SSC from diagnosis onwards. Although hardly anyone was familiar with SCPs, they perceived various potential benefits of SCPs, such as an increase in the patients’ self-management and providing HCPs with an up-to-date overview of the patient’s situation. Perceived preconditions for successful implementation included adequate personalisation, integration in the electronic health record and ensuring adequate funding to activate and provide timely updates.

**Conclusions:**

According to HCPs there is considerable room for improvement in terms of melanoma SSC. SCPs can assist in offering personalised and broader i.e., including psychosocial SSC. Aside from personalisation, efforts should be focused on SCPs' integration in clinical practice, and their long-term maintenance.

**Supplementary Information:**

The online version contains supplementary material available at 10.1186/s12885-023-10759-9.

## Background

The incidence of cutaneous melanoma has been steadily increasing and reached over 300.000 new cases worldwide in 2020 [1]. Novel therapies, including immune checkpoint inhibitors (ICIs) and targeted therapies (BRAF + MEK inhibitors), have led to an increased overall survival of patients with advanced disease [2, 3], converting metastasised melanoma into one of the first potentially curable cancers [4]. Consequently, more patients with melanoma are able to resume their lives, albeit with very variable prognoses, both within and between stages [5]. In-depth qualitative research has shown that metastatic melanoma patients have unmet needs in terms of survivorship care (SSC) such as the need for tailored information and broader supportive care [6]. Furthermore, even in patients with a thin (lower stage) melanoma *–* for whom the prognosis in most cases is excellent* –* the impact of the diagnosis is often significant and they need more SSC than currently provided [7].

SSC is defined as care provided to cancer survivors, focusing on prevention and identification of treatable cancer recurrences, second cancers, late effects and improving quality of life [8]. A central component of and a tool to provide SSC is a survivorship care plan (SCP), which aims at informing patients about the disease, treatment and its possible effects and improving coordination of care [8]. The recommended categories of SSC [8, 9] and components of SCPs [8, 10] are listed in Table 1. Providing adequate SSC can fulfil the unmet needs of cancer survivors including those with melanoma [6].

**Table 1:** Recommended categories of SSC and components of SCPs by the IOM [8]

Recommended categories SSC^a8, 9^	Recommended components of SCPs^b8, 10^
1. Information and education about the disease, its treatment and the possible early and late effects; 2. Identification and treatment of the disease and therapy effects on all possible domains (i.e. physical and psychosocial, including work- and insurance-related); 3. Oncological follow-up with surveillance for cancer progression, recurrences or second cancers; 4. Coordination between all the healthcare providers involved in the care process, to make sure all of the survivor’s health needs are met	• Cancer type, treatments received, and their potential consequences; • Specific information about the timing and content of recommended follow-up; • Recommendations regarding preventive practices and how to maintain health and well-being; • Information regarding employment and health insurance; and • Information on the availability of psychosocial, nutritional, and other supportive services

^a^SSC = Survivorship care, i.e. the care provided by either specialists or primary care providers to all cancer survivors, focusing on prevention, ensuring access to effective interventions and helping patients to improve their quality of life

^b^SCP = Survivorship care plan, i.e. comprehensive care summary and follow-up plan that is clearly and effectively explained and consists of critical information needed for the survivor’s long-term care

Despite the recommendation of the American Institute of Medicine (IOM) to provide all cancer survivors with an SCP [8], adopted by the Dutch melanoma guideline [11] as well as international (melanoma) guidelines [9, 11], implementation in clinical practice is limited [12, 13]. Only 5–52% of the healthcare providers (HCPs) provide their patients with an SCP [12]. When comparing cancer types, patients with melanoma appear least likely to receive an SCP [14]. These data, together with the reported unmet SSC needs, signal suboptimal provision and implementation of SSC and SCPs in melanoma care. Furthermore, little is known about how HCPs should provide SSC to patients with metastatic diseases with such varying prognoses as melanoma.

To optimise melanoma SSC, it is crucial to take into account the perspective of oncological HCPs, in addition to those of patients. However, how oncological HCPs view melanoma SSC and what they consider important regarding this topic has not yet been described in current literature. Therefore, the aim of the present study is to gain an in-depth understanding of the perspectives of oncological HCPs on appropriate melanoma SSC. This will enable tailored melanoma SSC to both patients’ and HCPs’ needs, which may lead to better implementation and effectiveness in clinical practice [8, 12, 15].

## Methods

### Study design and methodological considerations

A qualitative online focus group design was chosen as qualitative research is particularly suitable for in-depth exploration of experiences and perspectives on a particular subject [16]. Moreover, focus groups are expected to provide rich and diverse data because of the interaction between participants [17]. Due to the COVID-19 pandemic, we decided to organise online focus groups instead of face-to-face meetings to prevent unnecessary group gatherings. The reporting of this study followed the Consolidated criteria for Reporting Qualitative research (COREQ) [18].

### Setting and participant selection

This study was conducted as part of a project in which a personalised SCP for melanoma survivors will be developed. In this project, melanoma survivors are defined as individuals diagnosed with melanoma (stage I—IV) [10, 19]. However, ‘survivor’ and ‘patient’ are used interchangeably throughout this article. This project was performed in the region of Groot-Rijnmond with one participating academic hospital (Erasmus Medical Center) and four non-academic hospitals (Albert Schweitzer Hospital, Franciscus Gasthuis & Vlietland and Maasstad Hospital). Within this region, melanoma care is often multidisciplinary in nature and shared between specialists and primary-care providers [20]. For stage I follow-up is mainly GP-led, for stage II and III both dermatologist- and surgical oncologist-led and mainly oncologist-led for stage IV melanoma. This long-term care is covered by mandatory state insurance and if ongoing income is not covered by an employer, a sickness benefit provides a temporary income for these patients for a maximum period of 2 years [21].

To select participants, each participating hospital was asked to provide a list of all their HCPs involved in melanoma care i.e., dermatologists, surgeons, oncologists, oncological nurse practitioners, support counsellors and general practitioners (GPs). Eligible participants had to work within one of the participating hospitals or larger region and had to speak the Dutch language well. Potential participants received information about the study by email and could apply by filling in an online form (Additional file 1). They were offered a small thank-you gift for participation. Thirty-two HCPs were willing to participate. Based on their availability and using a purposive sampling method [22], 24 HCPs were invited to participate. The aim was having a variable sample in terms of age, sex, hospital type, profession and field of medicine in each of the focus groups. All participants signed a consent form. After four focus groups, varying from five to six HCPs per session, data saturation was reached i.e., no new themes were identified from the data [23].

### Data collection

Prior to the focus groups participants completed a short-self-administered questionnaire to collect demographics and received instructions on how to participate in the online sessions. The focus groups were held through Microsoft Teams® and moderated by at least two of four researchers, including a female medical doctor (N.K.), female medical student (R.B.), female psychologist (M.L.) and female dermatologist (M.W.). All participants knew the researchers’ background and reasons for doing the research. One researcher (N.K. or R.B.) took notes during the focus groups. The focus groups lasted 90 to 120 min. A topic guide, which was based on relevant literature [6, 8, 12, 15, 24], was used to structure the discussion (Additional file 2). Four main topics were addressed: perceived impact of melanoma on patients, current SSC/SCP practices, opportunities for improvements and perceived facilitators and barriers using SCPs. All focus groups were both audio- and video recorded.

### Data analysis

All recordings were transcribed verbatim in anonymised form. Video recordings were used to link statements to the correct participant. All transcripts were analysed using Nvivo version 12®. An elaborate thematic content analysis was performed consisting of several phases [25]: first, the researchers familiarised themselves with the data by rereading and summarizing each transcript [25, 26]. Subsequently, the transcripts were coded by one researcher (N.K. or R.B.) and then checked by a second researcher (N.K. or R.B.). During this initial coding process, the researchers identified all potential relevant themes [25]. The resulting unstructured list of initial codes was discussed with a third researcher (M.L.). In the second phase of analysis, the codes were sorted into a more structured coding list: relationships between all initial codes were identified and organised into main- and subthemes by two researchers (R.B. and N.K.). The resulting, structured list of candidate themes was discussed within the multidisciplinary research team (N.K., R.B., M.L.) until consensus was reached. In the final phase the list of candidate themes was reviewed, further refined and named (N.K., R.B., M.L. and M.W.), followed by checking them in accordance with the complete data set (N.K. and M.L.) [25].

## Results

### Participant characteristics

Characteristics of participating HCPs and group compositions are displayed in Table 2.

**Table 2:** Characteristics of focus group participants (n = 23)

Participant	Sex	Age	Hospital	Specialty	Frequency of involvement in melanoma care
*Focus group 1*					
HCP1	F	51	EMC	Support counsellor	Monthly
HCP2	M	53	EMC	Surgeon	Daily
HCP3	M	37	EMC	Oncologist	Daily
HCP4	F	33	EMC	Oncology nurse practitioner	Weekly
HCP5	M	50	-	General practitioner	Yearly
HCP6	M	58	MSZ	Dermatologist	Weekly
*Focus group 2*					
HCP7	F	37	EMC	Surgeon	Daily
HCP8	F	41	EMC	Oncologist	Daily
HCP9	F	55	EMC, MSZ	Support counsellor	Monthly
HCP10	F	43	MSZ	Surgeon	Weekly
HCP11	M	31	EMC	Oncology nurse practitioner	Daily
HCP12	F	41	ASZ	Dermatologist	Daily
*Focus group 3*					
HCP13	M	46	MSZ	Dermatologist	Weekly
HCP14	F	50	FGV	Surgeon	Weekly
HCP15	F	43	-	General practitioner	Yearly
HCP16	F	39	ASZ	Dermatologist	Daily
HCP17	M	55	ASZ	Surgeon	Weekly
HCP18	M	43	EMC	Surgeon	Weekly
*Focus group 4*					
HCP19	M	38	EMC	Dermatologist	Weekly
HCP20	M	44	ASZ	Surgeon	Weekly
HCP21	F	56	EMC	Support counsellor	Monthly
HCP22	M	57	FGV	Surgeon	Weekly
HCP23	F	52	-	General practitioner	Yearly

*F* Female, *M* Male, *EMC* Erasmus medical centre, *MSZ* Maasstad hospital, *ASZ* Albert Schweitzer Hospital, *FGV* Franciscus Gasthuis & Vlietland hospital

### Perspectives on appropriate melanoma SSC including SCPs

**Fig. 1:**
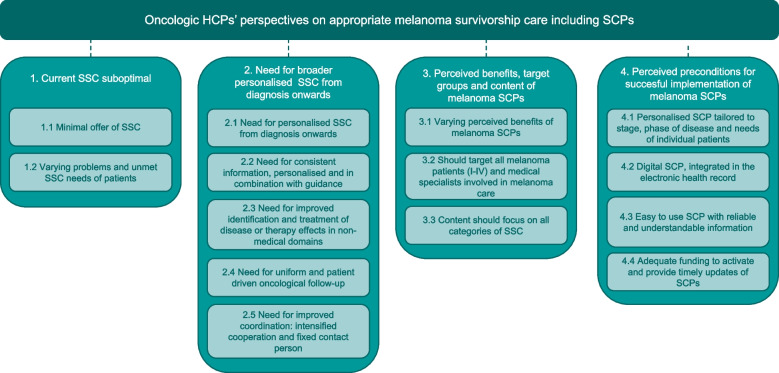
Overview of themes and subthemes

The analysis resulted in 4 main themes and 13 sub-themes (Fig. 1), which are discussed below.

### Current SSC Suboptimal

#### Minimal offer of SSC

HCPs regarded the current offer of melanoma SSC minimal and highlighted the difference with SSC offered to survivors of other types of cancer (e.g., breast- and colorectal cancer).

*I think it’s limited, especially compared to the SSC for other malignancies […] So no, I’m not that impressed by it [current melanoma SSC]. To be honest, my follow-up consults with melanoma patients never take very long.* – Surgeon, male, 57 (HCP22)

Furthermore, they considered current SSC mainly medically oriented, while optimal SSC should also include non-medical care and address psychosocial issues, such as work-related problems (see also 2.3).

### Varying problems and unmet SSC needs of patients

HCPs emphasised that melanoma can have a significant impact, but is variable among patients. For example, in stage I-II melanoma, some feel reassured after the excision, whereas others continue to feel afraid despite the melanoma being removed.

*I have always noticed a big difference when talking about the outcome of the excision. There are people who say: ‘Oh well, you cut it out, is there anything else we need to do or are we done?’ And there are people who think they’ll be dead by next week*. – Dermatologist, male, 38 (HCP19)

Consequently, unmet SSC needs vary largely among patients and therefore SSC should differ per individual, firstly by their disease stage, but also on setting of the disease, their prior knowledge about melanoma and their way of coping.

### Need for broader personalised SSC from diagnosis onwards

#### Need for personalised SSC from diagnosis onwards

Because of these varying needs, that may change throughout the disease trajectory, HCPs indicated different options of additional care and support should be offered, tailored to the patient’s needs. Whereas some patients are doing well and consider short check-ups sufficient, others need broader SSC.

Although the timing of the need for SSC varies among patients, HCPs indicated that challenges already can arise during the diagnostic phase, and therefore SSC should be offered from diagnosis onwards.

*I think SSC should start at the moment of diagnosis. And then, depending on how the patient reacts... some people will immediately need tools, answers and guidance. For others, that comes later.* – Dermatologist, female, 41 (HCP12)

Furthermore, HCPs mentioned that by adequately informing patients about their options and signalling their problems from the beginning, worsening of any occurring problems may be prevented. This in turn may result in healthcare cost reductions.

### Need for consistent information, personalised and in combination with guidance

According to HCPs, information provision related to SSC should be improved. They mentioned that because current (local) guidelines of all medical specialties involved in melanoma care contain different information regarding melanoma and especially follow-up, there is a lot of practice variation in information provision. HCPs emphasised this can cause stress among patients, and suggested multiple options for improvement: updating, adjusting and streamlining relevant guidelines, adequate continuing medical education (CME) for HCPs involved in melanoma care and centralisation of information. This could ensure that all specialties involved provide equal information, within one but also between different hospitals.

*The surgery guideline contains different information than the dermatology guideline. That is not acceptable. That creates confusion […] Practice variation causes a lot of stress for patients.* – Surgeon, male, 53 (HCP2)

Furthermore, HCPs stressed the importance of tailoring information to the individual patient: content of information should differ not only between, but also within all melanoma stages since even within one disease stage, treatments and prognoses can be different. Right after diagnosis, a standard leaflet with information about melanoma in general would be sufficient, while further along the patient journey patients should be informed accordingly. Therefore, they stressed information should be tailored to the patients’ disease stage, their specific situation and their individual needs.

Furthermore, since they noticed patients are often left with a lot of questions and concerns, they suggested to accompany information by actual guidance: since patients tend to forget a lot of the information received during the first and usually overwhelming consultation, they considered an extra appointment around one week after diagnosis useful for all patients. HCPs mentioned that, particularly for patients with lower stages (for whom follow-up usually consists of 1 follow-up check), putting more effort in adequate information provision around diagnosis, could prevent extra, unnecessary appointments at the dermatologist’s. Furthermore, sufficiently informing patients could prevent them from looking for (incorrect) information online. However, they indicated that it should be borne in mind that by offering patients a standard extra consultation, they can come up with many extra (unrelated) questions, which could take a lot of HCPs’ already limited time.

### Need for improved identification and treatment of disease or therapy effects in non-medical domains

HCPs agreed current SSC is mainly medically focused and pays too little attention to non-medical issues (see also 1.1). They stressed more efforts should be focused on psychosocial aspects including work-related problems. Such issues should at least be identified, for example by using short questionnaires or patient reported outcome measures (PROMs). Advantages of PROMs include signalling of patients’ problems, making them easier to discuss and encourage patients to prepare discussion points before the consultation. HCPs however, indicated they experience focusing on psychosocial topics as difficult, since they lack time to discuss topics as e.g., sexuality during consultations and do not know where to refer the patient if needed. The outcomes of these questionnaires would help them to refer patients to appropriate care if necessary.

*What I think, and notice among colleagues: they sometimes find it difficult to bring things up because they are afraid they’ll open some sort of cesspool […] of which they think: what do I do with this? […] it starts with identification, but you have to be able to do something about it. And I think that as long as practical tools to do something are lacking, we will never start identifying properly*. – Dermatologist, male, 38 (HCP19)

HCPs also indicated that patients rarely actively ask for extra support themselves, but also often do not know what their options and possibilities are. They indicated a referral guide could inform patients about these options and provide clarity about where to go with (both medical and non-medical) questions and complaints, strengthening patient’s self-management. Furthermore, they highlighted the potential role of support-counsellors in guiding patients to the right non-medical care and support. In addition, HCPs saw a potential role for the patient’s GP for more counselling, in view of their (closer) relationship with the patient.

### Need for uniform and patient driven oncological follow-up

Currently every HCP adheres to the (local) guidelines used in their hospital. Because these guidelines slightly differ per center, potentially causing unnecessary stress among patients, they emphasised the need for more uniform follow-up. HCPs stressed patients should be informed about their (expected) follow-up scheme to manage expectations. However, they also indicated the need for patient-driven follow-up where possible, adapted to the individual patient’s needs: they stated patients should be able to visit for skin checks at low threshold, as this contributes to regaining confidence in their skin.

Furthermore, they perceived moving the oncological follow-up of low-risk patients from specialists to GPs as an option. In addition, they argued that follow-up could be offered on indication instead of standard, since currently almost no recurrences or new tumors are found during scheduled follow-up checks of these patients. Additionally, according to HCPs, more and more patients prefer to perform these short follow-up checks either at the GP or from home, because hospital visits are often associated with disadvantages (e.g. time, travel distance and costs).

*Distance… transport… parking costs…. time… quite a few elements that people bring up to justify to prefer coming to us [GPs] for short consultations.* – General practitioner, female, 43 (HCP15)

However, not all HCPs agreed on moving oncological follow-up from secondary to primary care, because in their opinion not all GPs recognise skin abnormalities correctly, not all patients trust their GP and GPs might be too busy for this. The participating GPs agreed there is considerable variability of skills between GPs and moving this responsibility to them would have to be accompanied by proper training.

### Need for improved coordination: intensified cooperation and fixed contact person

Regarding coordination, HCPs stressed the importance of improving contact and cooperation between primary and secondary care, in which the GP should also play a bigger role. Although the cooperation between secondary and tertiary care is generally sufficient in melanoma care, they mentioned the feedback of information from tertiary to secondary care could be improved. Furthermore, they mentioned that patients now sometimes accidentally visit both the surgeon and dermatologist in one week, while that should be alternating. According to HCPs, one shared patient file would be the ideal solution.

Moreover, they mentioned that during follow-up the role division of all involved HCPs is clear, but not afterwards, in the period when the late effects occur. According to HCPs, region-wide agreements should be made with regional multidisciplinary rounds (MDRs) and a uniform and homogeneous care pathway (see also 2.4). They indicated melanoma care should be organised as one melanoma team. By working as a team and providing patients with a clear overview of the steps that will be taken, they can take away the patient’s uncertainty.

*I think that if […] you present yourself as a melanoma team, you can take away some of the patient’s uncertainty. So, you could say: “I'm the dermatologist, I've removed it, I've got bad news, but someone else from our team is ready and waiting to discuss the next steps with you... they know about you, they know what I've discussed with you and then you can ask your questions following this conversation”.* – Surgeon, male, 43 (HCP18)

Furthermore, they suggested a fixed contact person or case manager could give patients clarity where to go with questions. They considered a doctor’s assistant or a clinical nurse specialist (CNS) as options to fulfil this role, with the prerequisite that they must be properly trained.

### Perceived benefits, target groups and potential useful content of melanoma SCPs

#### Varying perceived benefits of melanoma SCPs

Almost all HCPs were unfamiliar with the term SCP and were unaware that an SCP is recommended in (Dutch) melanoma guidelines, yet they perceived several benefits of melanoma SCPs. First, it could ensure that all relevant information can be found in one place, which is convenient for patients and could also prevent them from seeking (incorrect) information online. Moreover, it could contribute to more responsibility and empowerment of patients and thus increase their self-management. In addition, HCPs also saw benefits for themselves: patients receiving the information they need may have fewer questions for the HCP during and in between consultations. Moreover, it could provide HCPs with a summary of the patient’s disease and (received) treatments. This ensures always having an up-to-date overview of the patient’s situation, even if for example, feedback of information failed.

*One advantage of course, could be that if a lot of HCPs can access it, you’d always be up-to-date with the latest developments […] I think if someone comes to my consult and I don’t have an up-to-date letter and they say, look at my app [SCP], that says this… that could be an advantage*. – General practitioner, female, 52 (HCP23).

### Should target all melanoma patients (I-IV) and medical specialists involved in melanoma care

HCPs believed an SCP should be provided to all melanoma survivors given the varying problems they may experience and the associated unmet SSC needs. However, they currently considered an SCP easiest to develop and implement for stage I to III melanoma given more uniform (follow-up) trajectories. Regarding stage IV melanoma, insufficient knowledge exists on standardised, uniform follow-up: HCPs were uncertain how follow-up e.g., after treatment with immunotherapy, should be organised, which could also vary from patient to patient. Since SSC needs start from diagnosis (see also 2.1), HCPs indicated SCPs should also be provided from that point on. In terms of HCPs, they believed that the target group should include all medical specialists involved in melanoma care i.e., the dermatologist, medical oncologist, surgeon and general practitioner.

### Content should focus on all categories of SSC

From the recommended categories of SSC (Table 1), HCPs considered several elements useful for inclusion in the melanoma SCP. Regarding category 1 they suggested including (links to) reliable information: about the disease and its treatment, but also on non-medical topics such as the potential psychosocial impact. The SCP should answer frequently asked questions about practical things and immediately refer to relevant information. Specifically for patients with stage III melanoma, HCPs suggested a decision aid for choosing or declining adjuvant therapy. In order to adequately detect problems of both disease and treatment (category 2), HCPs stressed that PROMS should be included in the SCP. In addition, the benefit of including a referral guide with contact information for support regarding both medical and non-medical problems was discussed. Concerning category 3, they suggested an overview of the patient’s (personal) follow-up schedule. Additionally, they suggested including a tool to take photos of skin abnormalities. According to HCPs, this could reduce fear among patients, facilitate early diagnoses and might be used for digital follow-up. Furthermore, enabling the patient to take and save photos themselves could also contribute to better cooperation between HCPs (category 4), as this would help them to distinguish between new and old (diagnosed by other HCPs) skin abnormalities.

*It would be nice if you can store photos in it […] ‘Where was it?’, ‘What did it look like?’, ‘Is this an in-transit metastasis or a new, second primary?’ That sort of things. It’s nice if the patient – some already do that – has the photos with them.* – Surgeon, female, 37 (HCP7)

In addition, HCPs indicated a tool to make (audio) recordings of consultations would be useful so patients can share them with close relatives to inform them about their disease, treatment and its (potential) impact.

### Perceived preconditions for successful implementation of melanoma SCPs

#### Personalised SCP tailored to stage, phase of disease and needs of individual patients

According to HCPs, the SCP must meet a number of preconditions in order to be successfully implemented in practice. First, melanoma SCPs should be personalised, whereby HCPs stressed they should not only be adapted to the disease stage, but also to the treatment trajectory the patient is in and tailored to individual needs.

*In terms of information provision, that’s different for each stage and even within a stage […] I think that each stage, each situation requires certain information.* – Oncologist, female, 41 (HCP8)

In order to really meet these individual needs, the patients should be involved in the process around the SCP – both in the design and the actual activation (see also 4.4) – and be able to decide for themselves if, and how they want to use it.

### Digital SCP, integrated in the electronic health record

Second, although currently a paper version of the SCP would still be convenient for people with limited digital literacy, HCPs agreed that a digital SCP would eventually work best in practice. Especially linking it to the electronic health record (EHR) would increase its use and effectiveness. Then, the patient would have all important information together at one location i.e., information about the patients’ disease and treatment, but also all other reliable (non-medical) information relevant to the patient (see also subtheme 3.2). Furthermore, linking the SCP to the EHR would make it easier (i.e., cost less time and effort) for HCPs to implement it in practice. Ideally, for example, if the HCP enters the stage and treatment in the patient’s EHR, this information should automatically appear in their SCP.

*If you could easily link it with the EHR […] and there you can assign something at the push of a button […] Then, during your consultations you can immediately click on something and say, okay, that needs to be added […] that would make it very easy.* – Oncology nurse practitioner, male, 31 (HCP11)

### Easy to use SCP with reliable and understandable information

As a third precondition, HCPs emphasised the SCP must be easy to use for both patient and HCP. It should be self-explanatory and activation of the SCP should cost as little time as possible. Furthermore, they indicated that one single tool for all diseases combined, would be the easiest to use for patients, as this would provide them with an overview of information and care for all their comorbidities.

In addition, the importance of incorporating reliable information that is of high quality, but at the same time easy to understand for the patient was emphasised.

*I think reliable and understandable information, easy to access and easy to work with.* – Oncologist, female, 41 (HCP8)

### Adequate funding to activate and provide timely updates of SCPs

Thinking carefully about the financial part of the SCP and making sure there is enough funding, was mentioned as a fourth precondition for successfully implementing the SCP. In addition, consideration must be given to who should activate the SCP; HCPs believed this should be very simple, preferably fillable by the patient him/herself. However, while they indicated the patient’s characteristics could be entered by the patient, HCPs stressed that entering the patient’s medical information cannot be done by the patient alone, as this could be dangerous (e.g., a patient might think he has stage IV when it is stage II). They stressed that either the patient’s specialist (i.e., dermatologist, oncologist or oncological surgeon) or CNS should provide help in this. Moreover, they stressed the importance of keeping the SCP up-to-date, because the content could quickly become outdated.

*Look at where we were with melanoma five years ago. It would of course be ridiculous if the app from five years ago was still in the app store. That would even be pretty dangerous*. – Support counsellor, female, 55 (HCP9)

In order to achieve timely updates, for which they stressed adequate funding is necessary, they indicated the information should be linked to existing information websites that are already regularly being updated. Moreover, also for keeping the content up-to-date they saw a role for the CNS.

## Discussion

This study reports HCPs’ perspectives on appropriate melanoma SSC including SCPs. The importance of personalisation was a central theme, both for SSC and SCPs. According to HCPs, melanoma SSC needs to be tailored to both characteristics (e.g., disease stage and type of treatment) and needs of individual patients. This fits the current trend towards personalisation of healthcare [27, 28] and aligns with previous research highlighting the importance of taking the individual patient' needs into account [6]. Research showed not everyone benefits from the same amount of information, where too detailed information could even have a negative effect on some patients [29]. Furthermore, as also indicated by melanoma survivors [6], HCPs stressed the need of focusing on broader SSC i.e., including psychosocial care. This is consistent with other studies both within [30–32] and outside the field of (skin)cancer [33], in which the need for non-medical care was emphasised. Our study is the first to show that HCPs share this view.

An important study finding is that HCPs perceive a potentially important role of melanoma SCPs in optimising SSC and tailoring it to individual patient’s needs. That is, when moving away from the current static, non-personalised (i.e., not needs-based) models [34]. They see its added value for patients as well as for HCPs themselves, especially in offering an (up-to-date) overview of relevant information on the disease and patients’ (medical) situation including e.g., diagnosis received care and schedule of follow-up. Giving patients more control over their health data has the potential to improve their self-management [36, 37]. Moreover, it allows them to share an up-to-date overview of their situation with other HCPs. The latter is particularly important in the multidisciplinary melanoma care and could improve coordination between HCPs [8]. Furthermore, an SCP can facilitate referring survivors to appropriate psychosocial care. HCPs in our study explained that identification of psychosocial problems will never be optimal as long as it is unclear where patients can be referred to. A practical locally adapted referral guide in an SCP could provide a solution for this.

Despite their promises, there are also several preconditions to be met to achieve successful implementation of SCPs. Aside from personalisation, HCPs stressed the importance of integrating the SCP in the EHR to facilitate its use in daily practice. By doing so, relevant patient data (like diagnosis and treatment) can be easily imported into the SCP and updated if the patients’ situation changes [38, 39]. Another important identified precondition is ensuring adequate long-term funding to activate, integrate and facilitate maintenance of the SCP. This aligns with previous studies emphasising the importance of sufficient organisational resources [40], as current SCPs are often not sufficiently integrated in care processes [38] thereby failing to ensure its continuity. To facilitate updating its content, HCPs in our study suggested (1) linking the SCP’s information to existing, reliable websites and (2) assigning someone, such as a CNS, to check on a timely basis whether its content is still in line with the current and constantly evolving medical knowledge and guidelines. This is consistent with previous research concluding that nurses are well-placed to provide education, care planning and support to cancer survivors [32, 41].

Our results showing that HCPs prefer a digital SCP, linked to the EHR, fits the current digital transformation of healthcare [42]. Although digital technologies such as a melanoma SCP, facilitate the delivery of personalised care [42], carefully addressing the variability in digital health literacy levels is warranted [43]. To develop an inclusive melanoma SCP, it is pivotal to tailor its content as well as adapt it to the needs of patients with lower levels of (digital) health literacy, and involve them in its development and implementation [43]. More research is needed on the most suitable ways of personalising digital technologies in (cancer) care to reach inclusive care.

Although they saw the potential value of SCPs, most HCPs were not familiar with the term SCP, let alone used it in practice [14]. More awareness on the existence of SCPs among HCPs as well as on the importance of providing adequate SSC is needed. This can be done by providing CME for all HCPs involved in melanoma care. Within this CME efforts should also be focused on working agreements in melanoma care to address the perceived practice variation and lack of uniformity. However, using an SCP as an HCP would probably reduce practice variation in itself, if it for example contains a uniform follow-up plan for that patient’s stage. In line with our finding that HCPs considered current follow-up for low-risk patients too frequent, previous research suggested a less-frequent follow-up schedule than currently is recommended in the Dutch guidelines as appropriate and safe [44] and a more patient-driven follow-up model should be considered when providing personalised SSC. As emphasised by the HCPs, further knowledge is required regarding the optimal organisation of follow-up care for stage IV patients.

To our knowledge, this is the first study providing an in-depth understanding of HCPs’ perspectives on appropriate SSC for patients with melanoma. We not only explored areas of needed improvement, but also provided suggestions for solutions. Previous studies investigating SSC were mostly aimed at patients with breast- or colorectal cancer, or cancer in general [12, 45]. Investigating this topic for melanoma is important as this group seems to lack proper SSC [6], despite increasing survival rates. By investigating the perspectives of a variety of HCPs, in addition to those of patients [6], a more broad, complete understanding on (needed improvement of) melanoma SSC was gained. As melanoma is one of the first metastatic diseases of which patients are starting to be considered cured after systemic treatment we believe our results can be used as blue-print for other metastatic diseases with a similar disease course (i.e., having substantially improved yet varying prognoses) and organisation of care to melanoma. In so doing, common challenges in implementing SCPs, such as adequate funding and updating its content, but also unique themes such as addressing the need for a personalised SCP, bearing in mind the prognosis switch [6], must be taken into account.

Due to COVID19 pandemic restrictions, online instead of face-to-face focus groups were performed. Whereas online focus groups could have been hindered by technical problems and by participants having insufficient digital skills [46], this did not occur, presumably as participating HCPs were already experienced in meeting online and were informed in advance. Although non-verbal communication in the online setting may not as easily be picked up as compared to face-to-face, the moderators felt they had a good overview, with all participants visible in one screen, and could therefore easily pick-up non-verbal cues and respond to them. Another advantage of the online setting was that it removed barriers such as travel distance and timing, making it easier to bring together a diverse group of professionals from different centers [47]. Since all participating HCPs were not familiar with an SCP, we were not able to describe barriers to its implementation like we intended. However, our results from these discussions provided valuable preconditions which can facilitate successful implementation. Finally, although this study is set within the context of the Dutch healthcare system, we believe the identified themes are transferable to other countries, especially to those in which melanoma care is organized in similar networks as is recommended in several guidelines.

In conclusion, according to HCPs, current melanoma SSC needs improvement and they emphasised the importance of offering personalised, broader (i.e., including psychosocial) care, which can be facilitated by (digital) SCPs. In addition to personalisation, inclusivity, integration in daily clinical practice, and long-term maintenance of the SCP need consideration. This will foster its implementation, long-term existence and use in clinical practice.

## Supplementary Information

**Additional file 1.**

**Additional file 2.**

## Data Availability

The data presented in this study are available on request from the corresponding author. The data are not publicly available due to privacy and ethical restrictions.

## Abbreviations

CME: Continuing medical education
CNS: Clinical nurse specialist
HER: Electronic health record
GP: General practitioner
HCP: Healthcare provider
ICI: Immune checkpoint inhibitor
MDRs: Multidisciplinary rounds
PROMs: Patient reported outcome measures
SCP: Survivorship care plan
SSC: Survivorship care
