# Supplementary material for: Oncological healthcare providers’ perspectives on appropriate melanoma survivorship care: a qualitative focus group study
Source: BMC Cancer. 2023 Mar 28;23:278. doi: 10.1186/s12885-023-10759-9 (PMC10042579; doi:10.1186/s12885-023-10759-9)
Supplement: Supplementary file 1 — Additional file 1. [file 12885_2023_10759_MOESM1_ESM.docx]

**Additional file 1 – Online application form**

In the first part of the online application form, potential participants were asked to provide the following demographics:

- Name
- Age
- Email
- Place of work
- Medical specialty
- Availability
- Address (to send the small thank-you gift)

The second part of the application form consisted of questions regarding their experience in melanoma care and their opinions on certain content areas:

- How often are you involved with melanoma patients (daily, weekly, monthly, yearly, never)?
- Do you think that current follow-up and supportive care for patients with melanoma is sufficient?
- Do you think there are areas for improvement for follow-up and supportive care of patients with melanoma?
- Are you familiar with survivorship care plans?
- Do you have any questions or remarks?
