# Supplementary material for: Oncological healthcare providers’ perspectives on appropriate melanoma survivorship care: a qualitative focus group study
Source: BMC Cancer. 2023 Mar 28;23:278. doi: 10.1186/s12885-023-10759-9 (PMC10042579; doi:10.1186/s12885-023-10759-9)
Supplement: Supplementary file 2 — Additional file 2. [file 12885_2023_10759_MOESM2_ESM.docx]

**Additional file 2 – Predefined topic guide**

Introduction

- Background and aim of focus group study
- Structure of the focus group
- Recording and anonymity

Part I: Survivorship care

Perceived impact of melanoma on patients’ lives

- Life domains
- Differences between (types of) patients
- Phases in disease trajectory
- Compared to other malignancies

Current practices in melanoma care

- Perceived content and definition of survivorship care (SSC)
- (Perception of) current melanoma SSC practices

Suggestions for improvement of melanoma SSC

- In general
- Following the four categories of SSC:
  - Category 1 - Information and education about the disease, its treatment and the possible early and late effects
  - Category 2 – Identification and treatment of the disease and therapy effects on all possible domains
  - Category 3 – Follow-up with surveillance for cancer progression, recurrences or second cancers
  - Category 4 – Coordination between all the health care providers involved in the care process, to make sure all of the survivor’s health needs are met

Part II: Survivorship care plans

General views towards survivorship care plans (SCPs)

- [Brief introduction to SCPs]
- Current use of SCPs
- [Extended explanation of SCPs incl. recommendation in guidelines]
- Perceived added value of SCPs
- Desired elements and functions of an SCP for patients with melanoma (e.g., format, starting point, needed level of personalisation)
- Perceived barriers and facilitators regarding SCPs for patients with melanoma
